# On the definition of chronic cough and current treatment pathways: an international qualitative study

**DOI:** 10.1186/1745-9974-10-5

**Published:** 2014-05-29

**Authors:** Shoaib Faruqi, Robert D Murdoch, Fabrice Allum, Alyn H Morice

**Affiliations:** 1 Department of Cardiovascular and Respiratory Studies, University of Hull and the Hull York Medical School, Castle Hill Hospital, Cottingham, UK; 2 Respiratory Discovery Medicine, GlaxoSmithKline, Stevenage, UK; 3 Global Market Research, Double Helix, London, UK

**Keywords:** Chronic cough, Definition, Management, Qualitative research

## Abstract

**Background:**

The pathogenesis of chronic cough is not well understood and treatment options are limited. In this study we sought to explore the current understanding and management of chronic cough across an international group of specialists.

**Methods:**

This was an international study of cross sectional qualitative design. In depth interviews were carried out with “Respiratory Specialists” experienced in treating treating Chronic Obstructive Pulmonary Disease (COPD), idiopathic pulmonary fibrosis (IPF), idiopathic chronic cough (ICC) and/or lung cancer patients and with “Disease Experts” in the field of Chronic Cough. Participants in the study were recruited from the USA, UK, Germany, Ireland, Australia and Japan. Interviews with specialists were held at research facilities and with DEs over the telephone. These were preceded by the specialists completing case records of patients recently seen. All interviews were conducted by native speaking trained moderators using a semi-structured interview guide script. This was designed to elicit the definition of chronic cough, explore the unmet needs for each disease state, define therapy goals, identify patient phenotypes and give an overview of the treatment pathway.

**Results:**

76 specialists and 10 experts took part in the study. Over two thirds (70%) of respondents defined chronic cough as “cough lasting more than 8/12 weeks” (range 2 weeks to 2 years). Physicians emphasised three interdependent aspects of clinical assessment: impact on quality of life, type of cough (productive versus non-productive) and the underlying pathology. Specialists emphasised treating the underlying cause rather than the cough, this being most prominent in Japan. Experts as a group focussed on chronic cough independently. Evaluation of the respiratory system, GI tract and upper airway (ENT) for establishing an underlying cause was recommended. Type of cough (productive vs non-productive) and impact on quality of life influenced treatment initiation. 33% of patients with ICC were prescribed anti-tussives. With associated diagnoses of COPD, IPF or lung cancer the emphasis was on treating the underlying condition. Alternatives to pharmacological treatments were frequently considered.

**Conclusion:**

There is significant international variation in our understanding and management of chronic cough. Further work is required to bring forth clear guidance and effective medicines for these patients.

## Background

Cough is the commonest symptom for which medical attention is sought, both in primary and secondary care and is the presenting symptom in more than half of all new referrals seen in the UK general practice [1]. The most common cause of acute cough is a viral upper respiratory tract infection. Chronic cough is a common accompaniment of several chronic respiratory conditions such as chronic obstructive pulmonary disease (COPD), lung cancer and diffuse parenchymal lung diseases. Persistent cough can also be a manifestation of non-respiratory conditions as well. It is difficult to quantify the exact prevalence of chronic cough as the response rates would depend on the question asked. It is estimated that chronic cough is reported in 3-33% of the population [2-4]. In our epidemiological study, chronic coughing, severe enough to interfere with normal activities of daily living, was reported by 7% of the general population [5]. There is considerable morbidity associated with chronic cough and the impact on quality of life is similar to other chronic lung diseases. Cough, therefore, has a considerable impact on the healthcare economy. For instance, the amount spent on over-the-counter (OTC) treatments for cough annually is in excess 400 million pounds in the UK [6]. Although cough is such an important problem the pathogenesis of chronic cough is poorly understood and therapeutic options are limited.

The most common aetiologies for chronic cough reported in literature are gastro-oesophageal reflux, eosinophilic airways disease and upper airways disease (rhinitis, post-nasal drip syndrome and sinusitis). However several series also report “idiopathic” chronic cough wherein no obvious cause can be identified despite extensive investigations [2-4]. This has been increasingly reported in recent series. Most patients with chronic cough exhibit a heightened cough reflex. This is exemplified by precipitation of cough by minor environmental stimuli, such as changes in temperature or on exposure to noxious stimuli such as tobacco smoke. It has been hypothesised that patients with chronic cough represent a distinct clinical entity consisting of chronic cough with this cough hypersensitivity. This has been termed the “cough hypersensitivity syndrome” and is being increasingly recognised as a diagnosis and suggests a mechanistic perspective to understand cough of any aetiology [7,8].

There has been an increasing interest in chronic cough recently with the publication of several international and national guidelines on this topic [9-16]. However many recommendations lack a robust evidence base and there are differences amongst these guidelines themselves. The way in which patients with chronic cough are investigated and treated may vary internationally. In this study we sought to explore the current understanding and management of chronic cough across an international group of specialists.

## Methods

This was an international, cross-sectional qualitative design study. Respiratory Specialists and “Disease Experts (DEs)” were identified who managed patients with chronic cough on a regular basis and in depth interviews were conducted with them. Prior to the interviews they were asked to complete Patient Case Records (PCRs). These specialists were identified on the basis of having experience in treating patients with Chronic Obstructive Pulmonary Disease (COPD), idiopathic pulmonary fibrosis (IPF), idiopathic chronic cough (ICC) and/or lung cancer (LC). Sampling framework included participants from four selected countries for specialists (USA, UK, Germany and Japan) and DEs (USA, UK, Australia and Ireland).

### Recruitment and eligibility criteria

All specialists were recruited by local specialist recruitment agencies with extensive experience in market research sampling. In order to ensure robust representation for each country, multiple locations within each country were chosen. US specialists were recruited and interviewed in three different locations; Baltimore, Dallas, and Chicago. Specialists from the UK and Japan were recruited and interviewed in two locations; London, Birmingham and Tokyo, Osaka respectively. German specialists were recruited and interviewed in 5 locations; Nuremberg, Essen, Fuerth, Erlagen, and Schwabach. To be eligible for inclusion in this study, specialists had to be practising in their specialty between 3–30 years, not being affiliated with any pharmaceutical manufacturer and not having participated in any COPD related research in the previous three months. Eligible participants spent at least 80% of professional time in direct patient care. All eligible specialists had to treat at least one of the following conditions: COPD, IPF, ICC or lung cancer. Specialists with primarily a COPD caseload had to have 40 or more COPD patients per month under their management at the time of recruitment, and their overall COPD caseload had to represent 15% or more of their chronic cough patients. Similarly, specialists with primarily a IPF or lung cancer caseload had to have 10 or more patients with IPF or lung cancer per month under their management at time of recruitment, and their overall IPF or lung cancer caseload had to represent 50% or more of their total chronic cough caseload. Finally, specialists with 10 or more patients diagnosed with ICC under active management at the time of recruitment were also eligible to participate in the study. Further to this, a sub-sampling strategy dividing the specialists’ sample according to caseload (IPF, ICC, LC or COPD), ensured that these were explored equally in detail at interview. Each condition was covered by a quarter of the respondents. At the end of each interview, participants were remunerated with a pre-agreed honorarium in their local currency.

Interviews with DEs were conducted over the telephone. To be eligible they would have to be practising in their speciality between 3–30 years and having an active chronic cough patient caseload (alone or as a manifestation of COPD, IPF, or lung cancer and minimum 15 patients per month). They would also be running chronic cough clinics regularly (minimum 8 per month). Eligible DEs must have authored/co-authored a recent peer-reviewed journal publication (within the last 3 years) in the field of chronic cough and presented a scientific paper in an international or national conference within the area of chronic cough. Additionally, DEs must have been principle investigators in a clinical trial within the field of chronic cough arena in the last 3 years and/or members on the editorial board of a peer reviewed journal publishing in this field.

### Study procedures & interview guide

**Figure 1:**
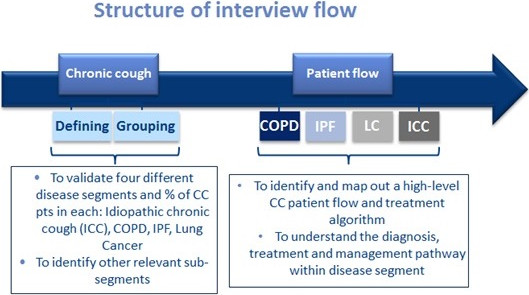
The structure and flow of the interview process.

The majority of specialist interviews were held at a research facility and were audio-recorded, videotaped and observed by the research team through a one-way mirror window at the facility venue. Interviews in Japan and Germany were conducted by native speaking moderators at the clinicians’ practice and were audio-recorded. For the DEs in-depth telephone interviews were audio-recorded. All interviews were conducted by moderators trained in qualitative methods. The moderators used a semi-structured interview guide script in order to elicit information meeting the purposes of this study. Specialists were asked to complete 3 PCRs prior to the interview with details from 3 recent patients seen with chronic cough (PCRs appended). Prior to the interviews, written informed consent was obtained by all participants. As this was a survey of physicians a formal ethics committee approval was not sought.The interview guide was designed to understand how chronic cough was defined by individual specialists, to explore the unmet needs for each disease state and define therapy goals as expected by them as well as to identify patient phenotypes and give an overview of the treatment pathway (field materials for specialists appended). Two separate guides were used for each group and both interview guides followed the same structure and content. The interview was introduced with a series of general questions around current role, practice and patient caseload. These were followed by questions to understand participants’ subjective definition of chronic cough. In order to maximise quantity and quality of information elicited, qualitative interactive exercises such as ‘natural grouping’ and ‘referral route mapping’ were also used. Laddering questioning on clinical factors included symptoms, frequency, duration and intensity of cough, type of cough and impact on quality of life. The final set of questions explored current patient management and the specialists’ role: understanding the treatment pathway and patient’s journey from diagnosis to treatment point, assessing current satisfaction with existing treatment choices and identifying pertinent unmet needs. The interview guide designed for the DEs included additional questions on future developments anticipated within the chronic cough field. Interviews with DEs focused exclusively on patients with a diagnosis of ICC and as they were telephone-based, use of interactive exercises was limited. The structure and flow of the interview process is shown in Figure 1.

### Data analysis

Results are from the qualitative discussions with the specialists and DEs as well as from completed PCRs. A thematic content analysis approach was adopted in order to analyse data from the interviews [17]. Interviews were transcribed from the native language and analysed by a qualified and experienced team of analysts. General themes for each area explored were identified and coded by an independent team of analysts, while the core team of researchers triangulated these, reconciling any coding disagreements. The saturated themes emerging from the final analysis are descriptively presented in line with the aims of the study. The definitions of quantitative notations such as “most/majority/some” are appended. Direct quotes reproduced from the respondents are in italics. In view of the differences between the interview focus between specialists and DEs direct comparisons between the two are not reported.

## Results

A total of 86 participants took part in the study of whom 76 were specialists and 10 were DEs. Thirty two specialists were recruited from the USA, 32 specialists from Europe (UK and Germany) and 12 specialists were recruited from Japan. Of the 10 DEs 3 were based in North America, 6 in Western Europe and 1 in the Asia Pacific.

### Chronic cough: caseload

**Figure 2:**
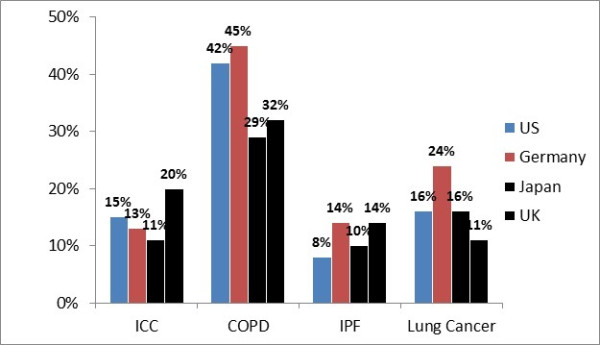
**Percentage of chronic cough case load ascribed to the different diagnoses is shown on the Y axis.** Self-completion form allowed for ‘other’ category and hence total does not sum to 100%.

**Figure 3:**
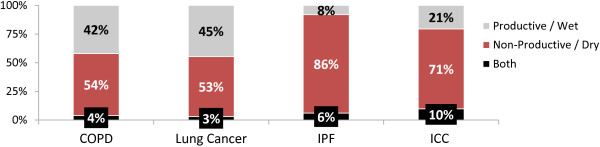
**This figure demonstrates the nature of cough, dry or productive, as a percentage in patients with COPD, lung cancer, idiopathic pulmonary fibrosis and idiopathic chronic cough.** Data was obtained from review of completed the patient case records.

The median number of patients with chronic cough seen by the specialists and DEs varied form 28 per month to 80 per month. The chronic cough caseload reported by US specialists was smaller (28 patients per month) than those reported from the UK (66 patients a month) and Japan (80 patients a month). However most US specialist straddled both an office and hospital based practice, unlike the other respondents. Median number of patients seen in a month reported by respondents in Germany and the DEs were 70 and 30 respectively. The aetiology of cough reported by the respondents is shown in Figure 2. COPD comprised a third to half of the case load of chronic cough. ICC case load as a diagnosis ranged from 11-20%. Self-completion form 1 was used to capture this data (see Additional files 1 and 2 for format).Whilst the majority of specialists perceived COPD to be associated with a productive cough, review of completed PCRs demonstrated a mixed picture. All specialists associated a dry cough with both IPF and ICC and the corresponding percentages for dry cough on review of patient records were 86% and 71% respectively. This is shown in Figure 3.

### Chronic cough: definition

**Figure 4:**
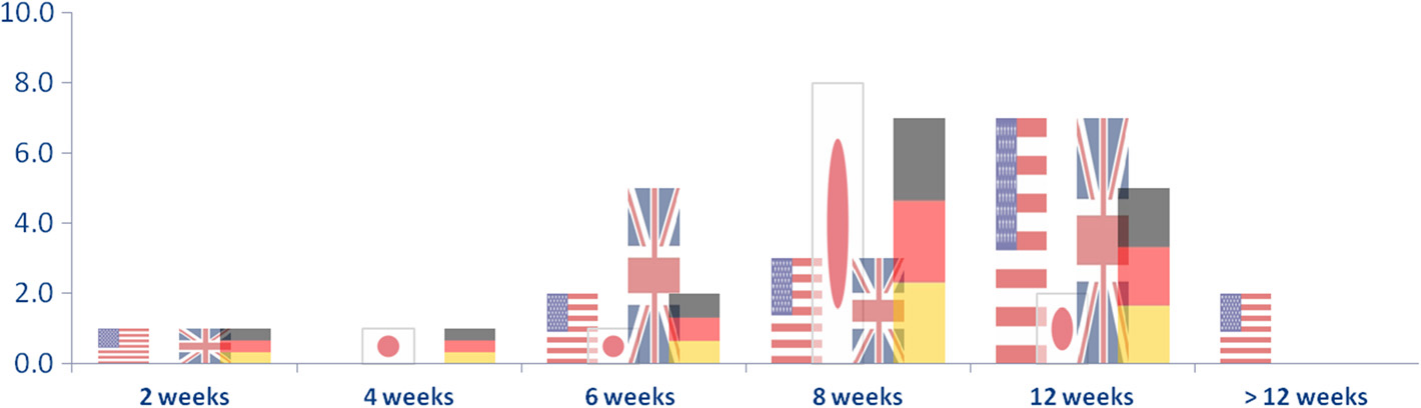
Physicians timescale of definition of chronic cough.

At the beginning of the interview, participants were asked how they define chronic cough in their daily practice and what factors they considered for this definition. The majority of responses revolved around the duration of chronic cough. The majority of responses defined chronic cough as “cough lasting more than 8 weeks” and “cough lasting more than 12 weeks”. A few specialists in the US suggested that definition encompassed presence of chronic cough for two consecutive years. When participants were prompted with a question about the current operational definition for diagnosing chronic cough, a general agreement across participants was observed. Diagnosing chronic cough was re-defined as “cough lasting more than 8 weeks” and Figure 4 shows the overall variance of responses for both DEs and specialists

“Put it this way if someone says I’ve got a cough and I’ve had it a few weeks, I always say well it will probably go away somewhere between 6 and 8 weeks and then after that you start thinking that it might be around for longer” (Specialist, UK)

### Chronic cough: impact on quality of life

Respondents thought currently available treatment for COPD was effective and were more satisfied with ability to address chronic cough in COPD than in other indications.

**Figure 5:**
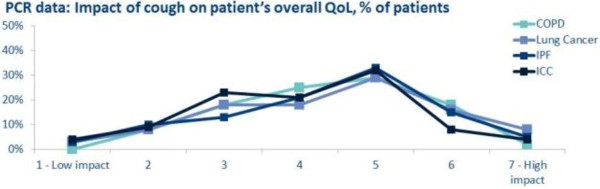
Perception of impact on quality of life of the various conditions causing cough.

*“Usually with the COPD patients that I have, when you have got them pretty tip-top medically treated, the cough is not usually a major issue.” (Specialist, US)*Opiates were widely used in patients with lung cancer and specialists were less worried about possible adverse effects. However the respondents perceived the risk-benefit ratio less acceptable in IPF and ICC, particularly in the group with ICC. Analysing the completed patient record forms, the impact of cough on quality of life was significant and very similar across all the conditions (Figure 5).

### Chronic cough: management and diagnostic grouping

**Figure 6:**
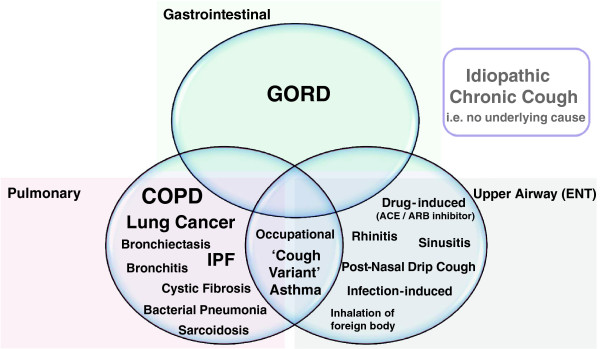
**The various diagnoses spontaneously reported by respiratory specialists as cause for chronic cough in their patients is shown.** The size script is weighted to reflect the frequency reported.

Various clinical factors were reported by specialists which influence management of a patient with chronic cough. The most influential factors reported were the underlying condition, the type of cough (wet, productive cough or dry, non-productive cough) and finally the extent to which cough impaired the quality of life of a patient. For the majority of physicians, when considering the underlying condition three medically relevant parameters defined patient grouping: respiratory, GI tract, and upper airway (ENT). Physicians further discussed how patients often had more than one condition which inevitably brought about other clinical factors into account to inform best treatment. The common diagnoses considered are shown in Figure 6.

“Some people don’t fit into one specific category; they often have multiple aetiologies for their cough” (Specialist, US)

“A lot of the underlying diseases are the actual cause of the chronic cough” (Specialist, Germany)

Another factor influencing treatment decision was the extent to which chronic cough impacted patients’ quality of life. Physicians assessed severity and interference of chronic cough with daily tasks, through their patients’ accounts on how disruptive chronic cough was and what impact it had in their daily life. Various factors were considered in the process of evaluating the impact of chronic cough in patients’ everyday functioning. A cluster of factors developed as the most indicative for estimating the practical and emotional impact that chronic cough has on patients’ quality of life and included: high cough frequency, presence (and frequency) of nocturnal coughing, working status (employed versus unemployed), activity status (active versus sedated lifestyle) and impact on social life (impaired social life due to chronic cough).

“An uncontrolled cough is very unpleasant for the patient.” (Specialist, Germany)

“The aspect of quality of life will come into it if there is a symptom that’s intruding on their quality of life enormously that will weigh in their decision of whether or not to take it.” (Specialist, US)

Looking at specific conditions, for COPD, the first line treatment was prescribing disease specific medications alone. This was explained by the expectation that as underlying pathology was being managed with targeted treatment, a secondary outcome would include alleviation of chronic cough. Initiation of symptomatic treatment for chronic cough was decided by the extent to which cough impacted quality of life. When chronic cough was highly bothersome and disruptive to the patient, cough medication was more readily prescribed in combination with COPD treatment upfront. However specialists in Japan would still not initiate any cough-specific treatment. In general, specialists were reluctant to initiate any cough-relieving medication to “wet cough” patients, although this opinion was less common in the US.

“The first thing I would do is treat them probably with an inhaled corticosteroid – treating the area of inflammation. If they have a lot of cough due to mucous production I might be giving them an anticholinergic inhaler as well, and then I might be giving them something specifically for cough or perhaps giving them an antitussive agent or hydrocodone or codeine cough syrup.” (Specialist, US)

For IPF patients, the severity of the condition drove treatment initiation. The lack of available targeted treatments meant physicians were more hesitant to initiate cough treatment unless symptoms were significantly bothersome to the patient.

“I ask the patient if the cough is unbearable. If they say yes, I will prescribe medication starting with a cough suppressant.” (Specialist, Japan)

“There are established treatment considerations for pulmonary fibrosis but our expectations of them working are relatively poor, we are a little bit more focused on symptoms in pulmonary fibrosis.” (Specialist, Germany)

For patients with LC, the priority was the most appropriate anti-cancer treatment. In palliative care settings cough relief was given more importance. However, cough-relieving medications were seen as a ‘last resort’ in end stage patients to maintain a level of comfort.

“Treating lung cancer should be top priority and the treatment directly influences patient prognosis. Cough often resolves when we succeed in shrinking tumour. Importance of managing lung cancer versus managing chronic cough for us is something like 80: 20.” (Specialist, Japan)

“The expectation is that the treatment itself (for the lung cancer) will in some way help the cough and it will go away so it’s obviously a problem then in the palliative setting when you’ve not really got any treatment to offer or any curative treatment to offer at the end, but patients are still troubled by cough, that can be tricky.” (Specialist, UK)

Similarly, the decision to give symptomatic cough treatment in patients with ICC was driven by the impact on the patient’s life. When chronic cough was significantly bothersome and disruptive, empirical treatment was more readily prescribed. However the emphasis was on establishing and treating an underlying cause first. Japanese specialists did not initiate any treatment for ICC, as it was less recognised as a condition itself but rather as a failure to diagnose an underlying pathology of a symptom. In addition to these, specialists discussed the limited options with regard to maintenance treatment for patients with ICC. As a result, specialists often prescribed alternative therapies to pharmacological treatments; some of these encompassed behavioural or physical techniques involving physiotherapist/speech therapist, whilst others spanned across antidepressants or tranquilisers and Chinese herbal medicines.

“Before we give someone a label that is idiopathic, we really want to be sure that we have excluded every other potential cause for cough so they will have had all of the tests and a CT scan as well.” (Specialist, UK)

### Chronic cough: differences in perception

Although specialists emphasised investigations some DEs felt there was not enough recognition of chronic cough as a disease state in its own right

“Wider respiratory specialist community were ‘hung-up’ on establishing an underlying cause”

“One of the reasons cough has not been pursued by industry is that there has been the perception cough is always due to something else. This has been the view that has been prevalent for the last 10–20 years and therefore has been very unhelpful.”

The label “Idiopathic chronic cough” was commonly utilised for patients in whom no underlying cause is found, however many other terms were also used. These include “unexplained cough”, “cause unknown”, “habitual chronic cough”, “psychogenic cough”, “cryptogenic cough”, “cough query cause” and “cough hypersensitivity syndrome”. Idiopathic chronic cough was widely recognised among specialists, except in Japan.

## Discussion

This international qualitative cross sectional study clearly demonstrates that isolated chronic cough and that associated with other chronic lung disease is an important problem with considerable associated morbidity. However significant variations were noted both in the understanding of chronic cough as well as its management. Lack of effective and safe anti-tussives was also highlighted.

Chronic cough has been arbitrarily defined as that which lasts for more than 8 weeks. Acute cough is defined as lasting less than three weeks. This distinction is important in clinical practice as the epidemiology and the aetiology of acute and chronic cough differ [14]. Most cases of acute cough are due to self-limited viral upper respiratory tract infections and resolve within this time frame. The cut off of eight weeks is used for defining chronic cough as it is thought that a cough lasting for longer than this is unlikely to be due to a simple respiratory tract infection, although recent ENT guidelines suggest a cut off of 12 weeks to define rhinosinusitis [18]. Often patients present with cough of a much longer duration and the range reported in various studies is quite large. Hence it is not surprising that there was a significant variation in response reported as to the understanding of the definition of chronic cough. Some respondents suggest a minimum duration of 2 years and this is likely to be based on a definition of chronic bronchitis wherein cough has to be reported for three months for 2 consecutive years [19]. We suggest that an agreed international definition of chronic cough is urgently required for epidemiology and appropriate management. It is also important in the context of research in this field to maintain a uniformity of inclusion criteria.

When reviewed in specialist cough clinics a cause or aggravating factor for chronic cough is usually identified in approximately 90% of cases with some clinics reporting a 100% diagnostic rate [20-22]. These series then report successful treatment outcomes on addressing the underlying cause. The three most common causes of cough identified in these series have been asthma and related syndromes, gastro-oesophageal reflux disease and upper airways disease (rhinitis, post-nasal drip syndrome and sinusitis) [2-4,20-22]. This and other similar “anatomic diagnostic protocols” have reported high success rates [23,24]. Hence it unsurprising that experts emphasised looking for an underlying cause and treatment of the same.

In epidemiological studies of cough the prevalence rates of individual diagnoses differ substantially. Gastro-oesophageal reflux disease, asthma and related conditions and post-nasal drip/upper airway disease account for 5%-70%, 10%-59% and 6% -93% respectively. Similarly a diagnosis of “idiopathic cough” has been reported in 0%-26% [2-4]. Thus, although “idiopathic chronic cough” was commonly recognised by most experts, it was not reported by any of the specialists from Japan. This could possibly be due to cultural differences in the willingness to accept an “idiopathic” diagnosis. Our multi centre international survey of over 10,000 unselected chronic cough patients shows a strikingly uniform demographic profile, with preponderance of middle aged and older females [25]. This increased female cough reflex sensitivity is associated with enhanced central processing of cough in healthy females. Irrespective of a possible aetiological factor we think that cough hypersensitivity is the cardinal feature of chronic cough. We suggest that a diagnosis of “cough hypersensitivity syndrome” is the best global descriptor for this condition. A recent survey of key opinion leaders by the European Respiratory Society indicated widespread acceptance of this term [26]. The use of cough hypersensitivity syndrome as an overarching label allows the inclusion of different clinical phenotypes within a spectrum of disorders causing cough.

Some disease experts thought that in the current paradigm there was too much emphasis on looking for an underlying aetiology rather than treatment of the cough itself. The reasons for these could be multi factorial and include geographical and perhaps cultural implications of treating a symptom rather than establishing the underlying diagnosis. This could be detrimental to the needs of the patient as often investigations are time consuming, invasive and expensive. We suggest that there is a need to regard chronic cough as a distinct clinical entity rather than exclude all possible underlying aetiologies. Empirical treatment for cough could be initiated and in parallel appropriate investigations organised to exclude serious pathology. There is an analogy to be had from the chronic pain field where similar to cough hypersensitivity, “hyperalgesia”, which also may have a central mechanism, is described [27]. In patients with chronic pain analgesics are usually promptly started and appropriate investigations organised concomitantly. However this approach in patients with chronic cough is limited by the current availability of proven, safe antitussives and may be a viable option in the future. Comparisons between current paradigms of management and of this approach could then be made.

A barrier to the empirical treatment of cough is the lack of efficacious therapies targeting afferent hypersensitivity. This was emphasised by most respondents in our survey, who would readily prescribe disease specific treatment, for instance for COPD, but reluctant to countenance the use of anti-tussives. We have previously described the efficacy of low dose morphine in patients with chronic cough [28]. Respondents would prescribe this in the palliative care setting but not otherwise, even in patients with severe COPD or end stage idiopathic pulmonary fibrosis where the prognosis may be similar to advanced lung cancer. Since the survey was conducted there have been further developments in this field with agents such as gabapentin being shown to attenuate cough [29]. In cough related to idiopathic pulmonary fibrosis thalidomide has demonstrated efficacy [30].

A limitation of this study is that is a questionnaire based survey of specialists and experts and only represents the views of the selected respondents, which reduces its generalisation. However the survey highlights the need for clear and internationally accepted guidance on the management of chronic cough. To recognise chronic cough as a distinct clinical entity would be useful both from the patient management and future research perspectives. We suggest that the current emphasis on diagnosis (often spurious) rather than therapy needs to change. There is a pressing need for the development of safe and efficacious therapy aimed at ameliorating cough hypersensitivity.

## Competing interests

Double Helix is an independent market research consultancy that was commissioned to conduct the primary market research. SF and AHM have no competing interests relevant to the study.

## Authors’ contributions

The study was commissioned and funded by GSK. The analysis of data was independent of GSK. RDM is an employee of GSK and holds company stock. SF drafted the initial manuscript which was subsequently reviewed and iterated by all the authors. All authors read and approved the final manuscript.

## Supplementary Material

Additional file 1 Patient case record. Click here for file

Additional file 2 Definitions of quantitative descriptions used in the manuscript. Click here for file
