# Supplementary material for: On the definition of chronic cough and current treatment pathways: an international qualitative study
Source: Cough. 2014 May 29;10:5. doi: 10.1186/1745-9974-10-5 (PMC4088926; doi:10.1186/1745-9974-10-5)

**Additional file 1: Patient case record**

FIELD MATERIALS

**Discussion Guide: 60 minutes
HCPs: Pulmonologist and Respiratory Specialists**

***1 GENERAL INTRODUCTION 3 minutes***

1. INTRODUCTION

- Thank respondent for agreeing to participate in interview
- Introduce yourself and company as an independent international marketing research company conducting studies on behalf of clients in the pharmaceutical field.

1. PURPOSE OF RESEARCH

- The purpose of this study is to discuss the current market landscape for chronic cough and to get your thoughts regarding the current patient management and treatment practices

1. CONFIDENTIALITY

Explain that:

- You and the company you represent are totally independent and you would like the respondent to be completely open and honest with their views.
- Everything is said in confidence - the respondent’s name will not be revealed to any other party
- This interview will last **1 hour**.
- Explain that tape/video recording is easier than note taking and allows free discussion. Explain that recording will ONLY be used for analysis of the interview, and enables us to observe the respondent.
- Any information shown during the course of this research should not be used to influence decisions outside the research setting
- You have the right to withdraw from the interview at any time during the interview process and to withhold information as you see fit
- Further, while they are being interviewed at the central location, there may be clients / colleagues behind the mirror observing the interview or viewing remotely via a video link.

**INTERVIEWER SAY:**

We are now being asked to pass on to our clients details of adverse events that are mentioned during the course of market research interviews. Although this is a market research interview and what you say will, of course, be treated in confidence, should you raise during the discussion an adverse event in a specific patient, we will need to report this even if it has already been reported by you directly to the company or the regulatory authorities using the MHRA’s ‘Yellow Card’ system.  In such a situation you will be asked whether or not you are willing to waive the confidentiality given to you under the Market Research Codes of conduct specifically in relation to that adverse event.  Everything else you say during the course of the interview will continue to remain confidential.

**Are you happy to proceed with the interview on this basis?**

**BEFORE STARTING THE INTERVIEW, PLEASE MAKE SURE RESPONDENT HAS BROUGHT THEIR 3 PCRs WITH THEM**

1. **WARM UP 2 minutes**

**Thank you for agreeing to take part in this interview. The discussion will focus on your experience of treating patients who experience chronic cough**

1. First of all could you tell me a little about your practice / where you work
   - Size of institution/ practice
2. How do you define chronic cough in your everyday practice?
   - What factors do you take into account when defining chronic cough?
   - What is your reaction to cough as defined as “lasting for more than 8 weeks”?

**BASED ON THIS DEFINITION OF CHRONIC COUGH ASK:**

1. And, thinking of your average caseload, how many patients with a chronic cough (including both new and repeat patients) do you personally see in a typical month?
2. ***PATIENT CLASSIFICATION/GROUPING 10 minutes***

**Now I’d like to ask you to complete a short exercise with me to help us understand your current grouping and management approaches for your chronic cough patients.**

**HAND RESPONDENT POST-IT NOTES**

Please write on separate post-it notes the initials (or anything else that uniquely identifies the person) of the last **6/7 patients** with chronic cough that you have seen. Please be assured that these interviews are entirely confidential so I do not want you to give me the patients’ full names, just an identifier for your own benefit.

Now I would like you to think about how you group your patient. Please arrange these 6/7 patients into **groups** using any **clinically relevant** criteria that you consider important in the management of these patients in your everyday clinical practice? Think about how you are currently treating these patients and the similarities / differences between them. **I do not want to talk about the different treatments that you use for these patients at the moment, just the patient characteristics that may influence this decision.**

**RESPONDENT TO SORT PATIENT POST-IT NOTES INTO GROUPS**

1. Please tell me how you have grouped these patients? Why?
   - What are the principle defining features of each group you have identified? **MODERATOR COVER BRIEFLY**
   - Why is this grouping clinically important? How are patients in each group managed differently?
2. What language do you use to describe these patient groups when speaking with your colleagues?

**MODERATOR RECORD PATIENT GROUPS ON SELF COMPLETION FORM 1**

1. What proportion of your chronic cough patient case load does each of these groups represent?

**DETERMINE PROPORTIONS OF EACH PATIENT GROUP AND RECORD ON SELF COMPLETION FORM 1**

1. Are there any other clinically relevant ways that you group your chronic cough patients?

**PROBE ON EACH THE FOLLOWING (esp. QoL):**

- - Underlying disease
    - What underlying diseases are present? (COPD, IPF, Lung cancer)
    - What proportion of your chronic cough patients have each of the following underlying diseases: COPD, IPF, Lung cancer and what proportion have Idiopathic Chronic cough?
  - Patient complains / does not complain of cough
  - Duration of cough
  - Frequency of cough
  - Intensity of cough
  - Well controlled vs. refractory
  - Ease vs. difficult to treat / particularly resistant
    - *If mentioned probe for specifics / examples*
  - Productive (wet) vs. non-productive (dry)
    - *Moderators note: the volume of sputum is key to classifying a “wet” cough as productive, technically it’s >25ml*
  - Time of day (nocturnal, during the day, both night and day)
  - Symptoms (i.e. wheezing, chest tightness, chest infection, Dyspnea)
  - Impact on QoL
    - i.e. Impact on patient’s day-to-day activities / physicians functioning, pain, general health / energy, social functioning, mental health/anxiety? Anything else?
    - Explore how physician measures and defines QoL in clinical practice

**REPEAT QUESTION 3.2 AND 3.3 FOR EACH OF PATIENT GROUP IDENTIFIED AND RECORD ON SELF COMPLETION FORM 1**

**IF NOT GROUPING PATIENTS BY UNDERLYING DISEASE ASK 3.5 and 3.6:**

1. To what extend is the underlying disease a concern to you in the proactive management of cough?
2. For the purpose of this exercise I’d like to understand what proportion of your chronic cough patients have each of the following underlying diseases: COPD, IPF, Lung cancer and what proportion have Idiopathic Chronic cough **RECORD ON SELF COMPLETION FORM 1**
3. ***PATIENT MANAGEMENT & CURRENT TREATMENT APPROACH 20 minutes***

**NOTE: USING THE SCREENER DATA PHYSICIANS ARE ASSIGNED TO A PATIENT TYPE: Idiopathic chronic cough (ICC) PATIENTS OR CHRONIC COUGH PATIENTS WITH ONE UNDERLYING DISEASE <COPD, IPF OR LUNG CANCER>. PHYSICIANS WILL FOCUS ON THE PATIENT TYPE THEY HAVE BEEN ASSIGNED TO FOR THIS SECTION OF THE INTERVIEW.**

**For this section of the interview I would like to focus specifically on your Idiopathic chronic cough (ICC) patients OR your chronic cough patients diagnosed with <COPD, IPF, LUNG CANCER> as their underlying disease**

**I would now like to understand in detail, the journey your ICC patients /chronic cough patients diagnosed with** **< COPD, IPF, LUNG CANCER> take from initially being diagnosed with chronic cough to how they are currently managed looking at the physician types involved in management and treatment approach taken.**

**Thinking about your ICC patient /chronic cough patient diagnosed with** **<COPD, IPF, LUNG CANCER> in general**

1. I would like you to please talk me through these patients’ management and treatment pathway detailing who was involved in the management of this patient and the treatment approach undertaken with these patients and why

- Please begin at initial presentation of the patient for chronic cough / initial chronic cough diagnosis up until on-going management
- What different types of HCP specialties were involved? and what role did these types of HCP specialists play?

**And with regards to their management and treatment**

1. Which **types of HCP specialists** did these patients see first for their chronic cough?
   - **(DO NOT ASK FOR HCPs ASSIGNED TO ICC):** To what extent did these patients present for their underlying condition versus their chronic cough specifically?
   - **(IF PATIENT PRESENTED FOR CHRONIC COUGH):** What triggered these patients to seek medical attention for their chronic cough?
     - And how long did they wait before seeking help?
2. Which HCPs usually **diagnose the chronic cough**?
   - How is the diagnosis made?
   - Tools/ tests/ exams/ symptom evaluation
   - And how typical is this diagnosis approach? What proportion of these patients are diagnosed in this way?
   - **(DO NOT ASK FOR HCPs ASSIGNED TO ICC):** To what extent is chronic cough diagnosed by the physician when consulting with a patient for their underlying condition versus part of an individual consultation initiated by the patient?
     - What barriers prevent patients from seeking help earlier than they do so?
3. What treatment are these patients taking for their chronic cough before they come to you?
   - Prescription treatment / OTT treatment?
   - What treatment would they have taken in the past?
   - If prescription treatment, who prescribed the treatment?
4. At what point did you become involved in the management of these patients?
5. When the patient presents to you, what do you do?
   - Diagnostic tests?
   - What treatment approach, if any, would you try next?
     - Rationale – positives, negatives
     - Duration of treatment
   - To what extent are the patients already receiving treatment for their chronic cough?
     - If so, how does this affect your treatment approach?
       - Continue treatment / Recommended or prescribed a new treatment?
   - How do you expect your treatment approach to change in the future for these types of patients?
6. Is there a time lapse between initial diagnosis and treatment initiation? If so, why?
   - If the patient presents without a confirmed diagnosis do you treat the cough or do you want for a confirm diagnosis before treating the cough?
   - **If physician treats the cough without an underlying diagnosis ask:** what is the 1^st^ line treatment? What, if any, subsequent treatments would you recommend/prescribe?

**Hand respondent SELF COMPLETION FORM 2: Patient flow Timeline**

1. To summarise what we’ve discussed I’d like to write it out on this patient flow timeline diagram. Please can you write in the typical patient flow for these types of patients **<ICC patients or chronic cough patients with COPD / IPF / LUNG CANCER>** on this timeline? You can see that we have started this off for you with ‘initial presentation to…’ and ‘on going treatment under…’ Please can you complete this by finishing these sentences (who does the patient initially present to and who managenes the on-going treatment) and then filling in the patient journey from initial patient presentation to on-going treatment above the line and time taken for each stage below the line (ie. number of weeks / months / years as appropriate)
   - Diagnostic tests? What tests conducted?
   - Diagnosis of chronic cough (above the line) – time period that lapse (below the line)
   - If present: diagnosis of underlying condition (above the line) – time period that lapse (below the line)
   - Patient managed by a different physician
   - Patient presented to respondent
   - Treatment
     - Patient taking treatment
     - Treatment prescribed by respondent

**IF UNDERLYING CONDITION PRESENT ASK:**

1. How important for you is managing chronic cough, compared to managing the underlying disease of the patient?

- Clinically important?
- What consequences, if any, result from not managing the chronic cough?
- Important with regards to Quality of Life / Work / Productivity?

1. If the underlying condition was to worsen (e.g. progresses from mild to moderate), to what extent would this cause the chronic cough to worsen?

- To what extent would worsening of the underlying condition increase patients complaints about their chronic cough?

1. What level of interaction is there between the HCPs involved along the pathway of these patients?
2. Who is ultimately responsible for the management and treatment decisions of chronic cough in this patient?
   - **PROBE** on role of respiratory specialist vs. acting consultant where necessary
   - What other factors influence the treatment decision?
     - Guidelines, clinical societies, hospital protocol?
   - And to what extent do you personally use treatment guidelines to inform your treatment decisions?
3. And who is responsible for ongoing management of this patient?
   - Is this a shared responsibility or is one physician responsible alone?
   - **PROBE** on role of respiratory specialist vs. acting consultant where necessary
   - How is any prior therapy managed between HCPs? To what extent will the HCP who assumes ongoing management discontinue previous drug treatment / star anew or build upon the prior treatment regimen?
4. Overall, on a scale of 1 to 10 (1 being not at all satisfied, 10 being highly satisfied) how satisfied would you say you are with current treatments available for this patient? Why is that?
5. What do you consider to be the greatest unmet need in terms of treating chronic cough in ~~this~~ these types of patients? Why is that?
   - What would enable you to meet this need? Why?

***Thank & Close***

**Adverse Event Reporting**

**IF RESPONDENT MENTIONS AN ADVERSE EVENT SAY:**

***"Doctor, [what you have just said]  / [what you said earlier in the interview] is classified as an adverse event.  The pharmaceutical company commissioning this market research has a legal obligation to report this as part of their ongoing benefit risk management.  I would like to spend a couple of minutes with you now to collect the necessary details of the Adverse Event, so that the pharmaceutical company can report this and meet their legal obligations.  Are you willing to assist with the reporting of this Adverse Event?"***

***IF YES: "Thank you.  The information you provide will be sent to the company's Drug Safety department who will contact you directly for further information.  Please note that if you provide your name during the Adverse Event reporting, this will not be linked in any way to your responses given during the interview."***

***Note: If respondent subsequently says no, then go to the ‘If NO’ section***

***IF NO:  "Because I have become aware of this reportable Adverse Event I am obliged to report this to the pharmaceutical company.  I will file this report without giving any of your details, but if the Drug Safety Department requires more information, may we contact you again (without identifying you to the pharmaceutical company)?"***

***IF STILL NO:          “Thank you doctor – I’ve made a note of that.”***

Example Patient Case Record Forms:


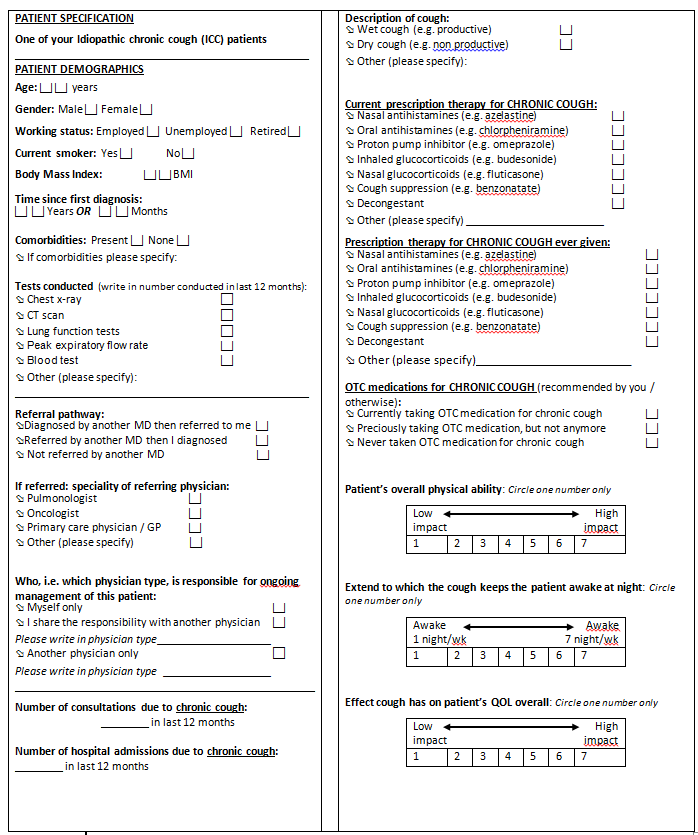


Self completion forms


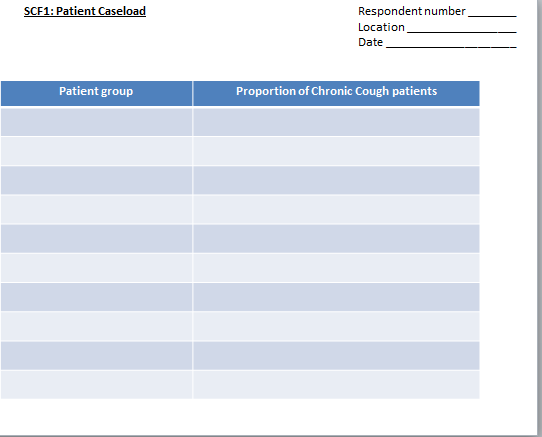


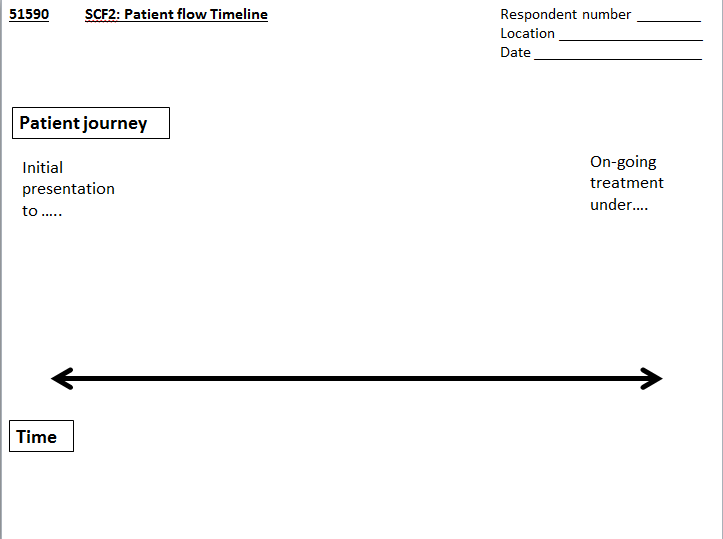


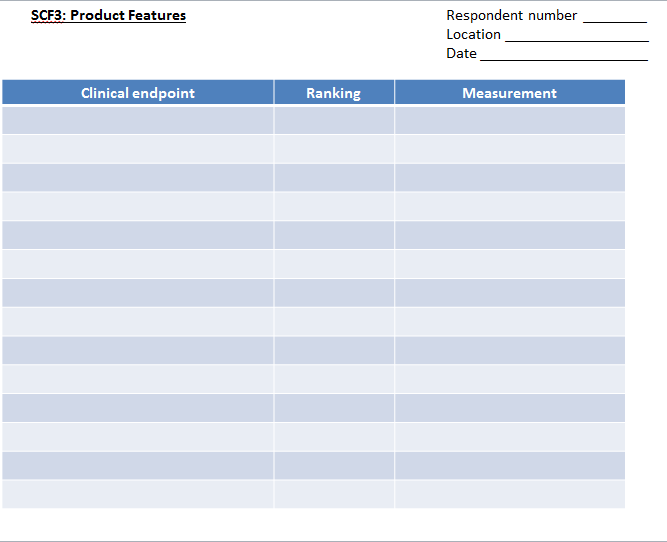

Supplement: Additional file 1 — Patient case record. [file 1745-9974-10-5-S1.doc]
