# Supplementary material for: On the definition of chronic cough and current treatment pathways: an international qualitative study
Source: Cough. 2014 May 29;10:5. doi: 10.1186/1745-9974-10-5 (PMC4088926; doi:10.1186/1745-9974-10-5)
Supplement: Additional file 2 — Definitions of quantitative descriptions used in the manuscript. [file 1745-9974-10-5-S2.doc]

**Additional file 2: Definitions of quantitative descriptions used in the manuscript.**

| **Notation** | **Approximate proportion of those questioned answering** |
| --- | --- |
| All | Unanimous |
| Vast majority | 80% + |
| Most | 70%+ |
| Majority | Over 50% |
| Half | ~ 50% |
| Some | Up to 50% |
| Several | A minority |
| A few | A small minority |
